# Predictive impact of different acute cannabis intoxication effects with regard to abstinence motivation and cessation of use

**DOI:** 10.1038/s41598-023-27592-6

**Published:** 2023-01-13

**Authors:** Thomas Schnell, Christina-Marie Grömm, Nils Klöckner

**Affiliations:** Medical School Hamburg, University of Applied Sciences and Medical University, Am Kaiserkai 1, 20457 Hamburg, Germany

**Keywords:** Psychology, Diseases, Medical research

## Abstract

Cannabis use is a common risk factor for psychoses. But although prevalence of consumption as well as potency of cannabis increased, the incidence of schizophrenia remained stable. The discontinuation hypothesis suggests that a potential increase of psychoses incidence may be relativized by more frequent cessation of consumption due to higher rates of adverse psychosis-like intoxication effects (PLE), caused by stronger cannabis. A mixed methods online survey was administered to 441 current and past users to analyze the predictive impact of different acute intoxication effects regarding abstinence motivation/cessation of use. Our hypothesis was that PLE would be experienced as the most aversive intoxication effect and therefore have the highest predictive significance. Possible confounds were included (craving, patterns of consumption and sociodemographics). Further analyzes compared past versus current users regarding the quality of intoxication effects, suggesting that past users retrospectively experienced more unpleasant experiences than current users. Free-text data explored subjective reasons for abstinence. We found that paranoid/dysphoric intoxication effects were most predictive for abstinence motivation. Less predictive were psychosis-like intoxication effects such as hallucinations. Group comparisons revealed significant more unpleasurable and less positive intoxication effects in past users compared with current users. Current users with the intention to stop consumption showed significantly more paranoia/dysphoria intoxication compared to users with no intention to stop use. As a conclusion, different intoxication experiences have different effects on abstinence motivation and substance use behavior. They therefore provide a focus that should be increasingly integrated into treatment concepts.

## Introduction

Psychotic experiences range from schizophrenia to psychotic-like experiences (PLE) in the general population without an underlying mental disorder^1,2^. Around 2–16% of the general population report lifetime PLE, such as hallucinations, delusions or depersonalization, usually transient or attenuated in nature^3^. Such experiences are associated with increased risk for psychosis^2^ and therefore may reflect an enhanced vulnerability^4^.

Cannabis users report various acute intoxication effects that range from relaxation to stimulation and from euphoria to anxiety. In higher doses and/or in vulnerable users, PLE may also occur in the context of acute intoxication^5,6^. According to preliminary estimates, cannabis users who experience PLE in the context of acute intoxication have an up to fivefold increased risk of developing psychosis compared to users without PLE experiences^7^. Among the latter, the risk of psychosis was similar to that of individuals who never use cannabis^7^. To explain the relation between cannabis and PLE, a complex interplay between neurobiological vulnerability of psychosis and cannabinoids or the endogenous cannabinoid system (ECS) is assumed^8,9^.

During the past decade, past-month prevalence of cannabis use increased by 27% in European adults up to 3.9%^10^, especially among younger people^11^. Concurrently, concentrations of delta-9-tetrahydrocannabinol (THC) in cannabis worldwide increased by 0.29% per year^12^. Such enhanced consumption of high-potency cannabis, considering complex interaction effects between cannabinoids and psychosis, leads to the suggestion that the incidence of schizophrenia will increase. Instead, incidence and prevalence of schizophrenia has remained quite stable^13^. To explain the unexpected stability of incidence rates, the discontinuation hypothesis is discussed^14^. Accordingly, high-potency cannabis is suggested to induce more PLE in users – and instead of continued use, which could be associated with a higher number of psychotic decompensations, increased adverse PLE-intoxication experiences would have led to increased cessation of use. Such discontinuation, especially in vulnerable persons with high sensitivity to cannabinoids may put a potential increase in incidence of schizophrenia into perspective^14^. Accordingly, it was confirmed that first-manifest schizophrenia patients who stopped consumption of cannabis subsequently had fewer psychotic relapses compared with patients who continued use^15^.

Thus, the central question is whether adverse intoxication experiences, and PLE in particular, lead to increased cessation of cannabis use, or at least to increased motivation to reduce use. Most previous studies on reasons for cessation of substance use or change motivation disregard acute intoxication effects. Continued use is often associated with social context, poor mental health, prior drug use and school failure^14^. Cessation is usually discussed as a consequence of increasing age and maturity^16^. As a psychological reason for maintaining substance use, craving is often considered as a predictor of relapse, frequency of use, and low abstinence rates^17^. In mental disorders, craving generally appears to be a factor maintaining substance use, according to the affect regulation hypothesis^18^. This is supported by the fact that substance use is generally more common in psychiatric patients than in the general population^19^. Accordingly, patients use drugs to reduce nonspecific dysphoric states. The corresponding type of craving is called relief craving, in contrast to reward craving, the hedonistic urge to use drugs^17^. The disregard of acute intoxication effects is also reflected by the fact that in German-speaking countries it was not even possible to operationalize these effects until the recent publication of a corresponding instrument^20^. However, in recent years, international research has turned to the subject, whose significance can be derived from the theory of operant learning: Accordingly, acute consequences of a behavior explain whether or not the behavior will be exhibited again, and the more immediately a reinforcement or punishment follows a behavior, the more predictively effective is the operant effect^21^. Pleasant acute intoxication experiences are accordingly associated with higher frequency of use, low abstinence motivation, and later dependence^22–24^. However, it is unclear whether the reverse is also true, that negative acute effects reduce or even terminate substance use behavior. In the case of addictive behaviors, additional neurobiological and psychological aspects come in to explain the maintenance of the behavior^25^. Accordingly, acute effects were found to be predictively relevant, particularly in the early stages of a substance use history, associating early positive, but not negative, acute use effects with continued heavy use^23,26^. At later stages of use, acute effects appear to be less effective. In summary, findings on adverse intoxication effects regarding consumption behavior are heterogeneous, ranging from decreased^27^ to increased use^24,28^. This heterogeneity may be explained by the fact that unpleasant intoxication effects represent a wide range of experiences from sleepiness, confusion and nausea to PLE. Experts suggest that especially PLE may be most aversive for users and PLE would have the greatest impact on consumers behavior^14^. In a recently reported study, authors confirmed their hypothesis that especially cannabis-induced PLE are associated with cessation of use^14^. If this finding can be replicated, this would be quite relevant, as close to 15% of users report such experiences^29^, which are also associated with increased risk of psychosis^7^.

Since abstinence motivation is a precondition for cessation of cannabis use, we first investigate the relation between different potentially predictors for stages of change motivation in a non-clinical sample. Of primary predictive interest are different intoxication effects. Our hypothesis is that aversive acute intoxication effects (and especially PLE) are associated with stronger abstinence motivation compared to positive effects. Based on the findings presented above, we also investigate the predictive value of sociodemographic variables, craving, and cannabis use parameters (frequency of use, duration of use and age at first use). Second, we compare three groups in terms of potential factors influencing cannabis use: Individuals who have stopped consumption, present users with intention to stop using, and present users without intention to change. Past users will be asked to retrospectively indicate their former consumption experiences. Our hypothesis is that aversive experiences of intoxication are found to be most pronounced among former users, followed by users with intention to change use. Regarding users without intention to change use, we hypothesize that positive intoxication experiences are more prominent.

## Methods

We developed a web-based version of the questionnaires used, referring to other research groups that use electronic versions in online samples and report acceptable psychometric properties^30,31^.

Our sample consisted of healthy people in the general population who volunteered to participate in the survey. Before the survey started, participants were explicitly informed by an introductory text that participation was voluntary and could be discontinued at any time. It was pointed out that only complete data sets would be processed. It was also emphasized that the data collection would be completely anonymous, i.e. no data would be collected and/or stored that could allow any reference to the individual person. We informed that the data would be used exclusively for scientific purposes. Finally, participants could only start the survey if they gave their written consent, by marking a corresponding field, placed at the end of the information page. This way, written consent was obtained from all participants included in the study. We did not obtain a further vote of an ethics committee in accordance with the regulations applicable in Germany, as we did not investigate a clinical trial with patients or any other vulnerable group.

### Sample selection and study procedures

Quantitative data were collected online using three self-report questionnaires via the survey site Unipark. In addition, qualitative data were collected using free-text fields, for a deeper understanding of abstinence motivation/reasons to stop cannabis use. The survey took place between November 2020 and April 2021. Recruitment of participants was performed by advertising the study via public notices. In addition, the link to the survey was sent out via social media (Facebook, Twitter, Reddit) and relevant cannabis forums. Inclusion criteria were that participants were German-speaking adults (minimum age of 18 years) and that they had previously used cannabis. No reward in form of an incentive was given.

### Survey questionnaires/measures

#### Sociodemographic data, substance use data and mental health

Information on sociodemographics included age, gender, education level and occupation status. Lifetime diagnosis of a mental disorder was recorded as self-report using a dichotomous item (yes/no; wording of the question: “*have you ever been diagnosed with a mental disorder?*”). Cannabis use patterns were collected, i.e. age at onset of use, time since last dose (days) and abstinent weeks within the last year, duration of regular use (month) and average frequency of use (joints per month). Regarding the latter, past users were asked to estimate their average use at the time of active use. The use of substances other than cannabis was also recorded by means of a dichotomous item (yes/no).

#### Cannabis intoxication effects (CanTox-17)

CanTox-17 is the German version of the original Cannabis Experiences Questionnaire (CEQ). Developed initially by Barkus and colleagues^32^, different English-language factor solutions have been subsequently published^31,33,34^. The German version was developed by our group^20^ and it consists of three negative dimensions and one positive dimension of acute cannabis intoxication effects: (1) “psychotic-like experiences/loss of reality” (feeling threatened, hearing voices, losing ones sense of reality, feeling like going crazy); (2) “paranoia/dysphoria” (feeling paranoid, anxious, nervous, depressed); (3) “confusion/disorientation” (speech becoming slurred, time slowing down, disturbed in thinking, not aware of what was going on); (4) “euphoria/creativity” (understanding the world better, feeling energized, full of ideas and more creative). Items were scored on a Likert scale with five graduations. Since CanTox-17 was just developed and evaluated within in a recent study^20^, the presented factor structure is to be considered as preliminary result. So, we again analyzed and replicated sufficient goodness-of-fit indices of the instrument and the internal consistency of the factors (cronbach´s alpha). Since the χ^2^-test is considered to be sample sensitive, the ratio between χ^2^- and the degrees of freedom was formed (χ^2^/*df*), with good to acceptable fit that is suggested to be 0 ≤ χ2 /df ≤ 3^35^. A χ^2^/df value of 1.91 in the previous evaluation study and 2.81 in the present study represent a good to acceptable fit. The root mean square error of approximation (RMSEA) < 0.08 indicates satisfactory model fit. The respective value of 0.058 within the evaluation study and the present value of 0.064 are in line with this criterion. In accord with Browne and Cudeck’s and Vandenberg and Lance criterion^36,37^, the comparative fit index (CFI), and the Tucker Lewis index (TLI) each > 0.90 also indicate a satisfactory model fit. The preliminary evaluation study identified a CFI = 0.93 and TLI = 0.92, compared with CFI = 0.92 and TLI = 0.91 in the present study. To determine the internal consistency of the four factors, Cronbach’s alpha was applied^38^. Within the previous evaluation study, values ranged between 0.74 and 0.78, whereas the present data reveal values between 0.70 and 0.79, representing an acceptable to good internal consistency.

#### Craving parameters

Cannabis craving was assessed using the Cannabis Craving Screening-7 questionnaire (CCS-7)^39^. CCS-7 consists of seven items, three items for the (1) “reward dimension”, and four items for the (2) “relief dimension” of craving. Participants had to rate each item on a Likert-scale with seven graduations. Various studies indicate that these dimensions can be reliably measured with the CCS-7^17,39^.

#### Stages of abstinence motivation

Motivation for abstinence was determined by using the SOCRATES (Stages of Change Readiness and Treatment Eagerness Scale) questionnaire consisting of 19-items related to three subscales, each capturing different aspects of patients’ motivation. These are (1) “ambivalence” (indicator for the process of balancing two options—namely, to remain or become abstinent versus continued substance use), (2) “recognition” (indicator for awareness of problem), and (3) “taking steps” (indicator for the concrete efforts to remain abstinent)^40^, Participants rate each item on a likert-scale with five graduations. In their assessment of psychometric criteria of the original version, authors conclude that SOCRATES questionnaire can be considered promising^41^. The instrument was subsequently used many times in empirical investigations.

#### Qualitative data on reasons to stop cannabis use

Qualitative methods (free text input fields in the questionnaire) were used to collect the specific reasons of past cannabis users that led them to stop consumption.

### Statistics

First, preparatory analyzing ensured the quality of applied instruments and managed missing values. Analyzing missing values revealed a proportion of missing values of 0.71% (minimum value = 0.2%; maximum value = 2.6%) at the case and variable levels. To analyze random occurrence of missing values, Little’s MCAR test was performed. Values of the questionnaires did not reveal significance as predicted. Consequently, missing values could be imputed using the expectation–maximization method. Respective CanTox-17 questionnaire, we analyzed goodness of fit criteria (see section above).

Second, we aimed to identify predictors of abstinence motivation. In a first step, we correlated potential predictors with each of the three stages of SOCRATES change motivation. Following findings of other groups^14^, key potential predictors were different cannabis intoxication effects, but also sociodemographic parameters such as age and gender. In addition, as craving is considered a key indicator of consumption relapse, both relief and reward craving were included in the analyzes. Finally, we analyzed specific cannabis use patterns. Those potential predictors that correlated significantly with motivational stages were then integrated into multiple stepwise regression analyzes with SOCRATES-dimensions as criterion (dependent variables). Cohen’s *f*^2^ was calculated as the effect size^42^, with a value of 0.02 corresponding a small effect, 0.15 representing a medium effect, and 0.35 representing a strong effect.

Third, complementing findings on abstinence motivation according to SOCRATES-data and following previous findings of expert colleagues^14^, we compared acute and past cannabis users. We divided the sample into three groups: Group 1 integrated acute cannabis users without intention to quit. Group 2 consisted of acute users with intention to discontinue cannabis use, and group 3 consisted of individuals who had already discontinued consumption. Descriptive analyzes and significance tests examined whether the groups differed significantly with respect to sociodemographic parameters, i.e., whether they were comparable with respect to the target parameters. The latter focused on group comparison with respect to acute cannabis intoxication effects, craving, and cannabis use parameters. Finally, we compared groups using multiple analyzes of variance (MANOVAs) including post-hoc analyzes with pairs of groups. To indicate the effect sizes, *partial eta*^2^ was calculated.

Quantitative analyzes were complemented by qualitative (free-text) information provided by past users in the questionnaire on their reasons for discontinuing consumption. Statements were transcribed, collated and inductively coded by an independent researcher. According to the thematic approach^43^, themes were derived naturally from the data. Second, instead of an a priori coding structure, themes were grouped in meaningful ways. This was followed by content interpretation of data. Results were discussed and verified within team meetings of our group. If the interpretation of the items differed among group members, discussions were held with the group until a solution was found. Experienced psychological psychotherapists with a high level of clinical expertise, especially with psychotic disorders and substance use disorders, were always present at the discussions.

### Ethics approval

All procedures of the study were carried out in compliance with the latest revision of the Declaration of Helsinki.

## Results

### Demographics and cannabis use patterns

Descriptive data of our sample regarding the parameters collected are presented in Table 1.

**Table 1:** Descriptive date of the sample.

	*N*	*M*	*SD*
Age	441	24.24	5.55
Gender (male/female)	235/205		
Level of education^a^	7/29/177/163/65		
Employment (employed, in training/unemployed)	395/46		
Mental disorder diagnosis (yes/no/unknown)	66/333/42		
**Cannabis use parameters**			
Age at onset (years)	441	15.93	2.49
Time since last dose (days)	441	185.81	712.23
Abstinence time last year (weeks)	441	20.61	18.31
Average frequency of use (units per month)	441	21.66	56.51
Duration of regular use (month)	441	26.27	47.61
Use of other substances (yes/no)	348/93		
**Motivational stages**			
Intention for abstinence (no/intention/cessation)	150/186/105		
Actual use of cannabis (yes/no)	336/105		
SOCRATES: Taking steps	441	2.36	0.95
SOCRATES: Ambivalenz	441	2.52	1.08
SOCRATES: Recognition	441	2.05	0.89
**Cannabis intoxication effects**			
CanTox: Psychosis-like/loss of reality	441	1.56	0.64
CanTox: Paranoia/dysphoria	441	2.26	0.78
CanTox: Confusion/disorientation	441	2.97	0.77
CanTox: Euphoria/creativity	441	3.40	0.78
**Craving**			
CCS-7: Reward craving	441	3.87	1.99
CCS-7: Relief craving	441	2.58	1.44

^a^Elementary school degree after grade 9/intermediate school degreee after grade 10/A-level grade 13/University degrees/Apprenticeship; CanTox, Cannabis-intoxication questionnaire; CCS-7, Cannabis Craving Screening with 7 items; SOCRATES, Questionnaire assessing motivational stages; *N*, frequency, *M*, mean value, *SD*, standard deviation.

### Cannabis intoxication effects and motivational stages (change motivation)

To identify potential predictors for different motivational stages (SOCRATES scales), we first investigated associations between motivational stages and potential predictors using correlative analyzes. Following previous findings by other groups^14^, we correlated motivational stages with cannabis intoxication effects, with craving as a key factor explaining consumption relapse, and also with frequency of cannabis use, age at onset of use and sociodemographic data. Parameters that significantly correlated with amotivational stage were integrated into multiple regression analyzes. Table 2 presents the significant predictors for each of the three motivational stages, as well as parameters included in the analyzes but not significantly predicting the criterion (the latter listed under “excluded parameters” in Table 2).

**Table 2:** Multiple regression: predictors of motivational stages.

	B	SE	*β*	T	Sig
**Taking steps**					
1. CanTox paranoia/dysphoria	0.422	0.071	0.305	5.974	≤ 0.001
2. Gender^a^	0.248	0.098	0.13	2.534	0.012
3. Abstinence time last year	− 0.008	0.003	− 0.125	− 2.428	0.016
R^2^ 0.139					
Corr. R^2^ 0.131					
F (df = 3; 332) 17.843					
Two excluded parameters: CanTox psychosis like/loss of reality; age at onset of cannabis use					
**Recognition**					
1. CanTox paranoia/dysphoria	0.311	0.075	0.239	4.142	≤ 0.001
2. Abstinence time last year	− 0.014	0.003	− 0.236	− 4.912	≤ 0.001
3. Relief-craving	0.126	0.03	0.201	4.225	≤ 0.001
4. Gender^a^	0.371	0.083	0.207	4.481	≤ 0.001
5. CanTox psychosis-like/loss of reality	0.283	0.098	0.167	2.885	0.004
R^2^ 0.310					
Corr. R^2^ 0.300					
F (df = 5; 330) 29.682					
Five excluded parameters: CanTox confusion/disorientation; age at onset of cannabis use; reward-craving; average frequency of cannabis use; duration of regular use					
**Ambivalence**					
1. Abstinence time last year	− 0.024	0.004	− 0.333	− 6.759	≤ 0.001
2. Gender^a^	0.452	0.098	0.209	4.617	≤ 0.001
3. Reward-craving	0.131	0.029	0.222	4.541	≤ 0.001
4. CanTox paranoia/dysphoria	0.239	0.077	0.152	3.094	0.002
5. CanTox confusion/disorientation	0.192	0.073	0.129	2.616	0.009
R^2^ 0.342					
Corr. R^2^ 0.333					
F (df = 5; 330) 34.376					
Six excluded parameters: CanTox psychosis like/loss of reality; CanTox euphoria/creativity; age at onset of cannabis use; relief-craving; average frequency of cannabis use; duration of regular use					

CanTox, Cannabis-intoxication questionnaire; Sig, level of significance;

^a^Male gender predicting dependent variables.

Regarding cannabis intoxication effects, paranoia/dysphoria in particular emerges as relevant positive predictor of motivation to change. At the highest and the middle motivational stage, it is in each case the strongest predictor. In addition, the remaining two unpleasant acute effects are also significant as positive predictors in the middle and lowest stages of change motivation. Pleasant cannabis acute effects, on the other hand, are not predictive of change motivation.

It is also interesting that hedonistic reward craving is positively associated with the low motivational stage of ambivalence, whereas relief craving acts as a positive predictor at the next-highest stage of recognition. The highest motivational stage (taking steps), where steps of change have already taken place, is not predicted by craving anymore.

All three regression models include the variable gender as a significant predictor. In each case, the male gender predicts the outcome parameter. To provide arguments for the subsequent discussion of this finding, we retrospectively analyzed male and female users regarding potential differences in patterns of cannabis use and acute intoxication effects. We found significant differences in the average duration of regular use (males: 33.83 months with a standard deviation of 60.06; females: 17.70 months with a standard deviation of 24.71 (*p* ≤ 0.001)). Further differences were found in the average abstinence time within the last year (males: 18.33 weeks with a standard deviation of 17.42; females: 23.10 weeks with a standard deviation of 18.96 (*p* = 0.006)). Regarding acute intoxication effects, only positive effects (euphoria/creativity) differed with a higher mean average for men of 3.52 and standard deviation of 0.68, and a lower mean average for women of 3.26 and standard deviation of 0.87 (*p* ≤ 0.001).

In addition, all regression models include the number of abstinent weeks as a negative predictor for motivation to change. Thus, the more regular the use, the stronger the desire to change. This is most pronounced in the motivational stage of ambivalence, followed by recognition, and the predictive value is lowest in the highest motivational stage.

Effect size calculations for “taking steps”: The effect size *f*^2^ according to Cohen^42^ is 0.143 for the overall model, which corresponds to a medium effect. Regarding individual predictors, the intoxication effect “paranoia/dysphoria” represents an almost medium effect (*f*^2^ = 0.103). The other two influencing factors have only a very small effect (each *f*^2^ = 0.02).

Effect size calculations for “recognition”: The overall model reveals a medium to strong effect with *f*^2^ = 0.24. Individual predictors range between small to medium effect sizes *f*^2^ between 0.05 and 0.07, while the acute intoxication effect “psychosis like experience/loss of reality” has a small effect with *f*^2^ = 0.02.

Effect size calculations for “ambivalence”: The effect size for the overall model is *f*^2^ = 0.3, which almost corresponds to a strong effect. With regard to the individual predictors, small to medium effects can be observed in each case.

### Group comparison of current versus past users

We compared three groups: Current cannabis users were divided into two groups (with and without intention to stop cannabis use), past users were the third group. Multivariate analyzes of variance (MANOVA) and *Chi*^2^-tests revealed no significant group differences regarding sociodemographic data except for age (which, however, was not found to be predictive with respect to the target parameters), employment status, diagnoses with mental disorder (self-report) and age at onset of cannabis use.

Significant group differences were identified in all acute cannabis intoxication effects and both variants of craving. Further, average frequency of cannabis use was about twice as pronounced in the group with current users (with intention to stop consumption), compared with the other groups.

Table 3 displays group differences.

**Table 3:** Group-comparison of current (with and without intention to stop use) and past cannabis users.

	Current users, no intention to stop use (*n* = 150)	Current users, intention to stop use (*n* = 186)	Past users (*n* = 105)	df	F/Chi^2^	Sig
Age (*M* (*SD*))	24.46 (6.57)	23.24 (3.54)	25.71 (6.5)	2	7.02^a^	**0.001**
Gender (*n* = m/f)	73/77	106/80	56/48	2	2.32^b^	0.313
Employment^c^	139/11	167/19	89/16	2	4.14^b^	0.126
Mental disorder^d^	15/119/16	28/138/20	23/76/6	4	8.42^b^	0.077
Age at onset (years)	16.15 (2.03)	15.6 (2.08)	16.19 (3.49)	2	2.86^a^	0.058
Average frequency of use	14.85 (25.10)	30.92 (78.69)	14.98 (36.42)	2	4.39^a^	**0.013**
CanTox: Psychosis-like/loss of reality	1.38 (0.52)	1.51 (0.53)	1.89 (0.83)	2	22.75^a^	**≤ 0.001**
CanTox: Paranoia/dysphoria	1.92 (0.66)	2.31 (0.65)	2.64 (0.91)	2	31.74^a^	**≤ 0.001**
CanTox: Confusion/disorientation	2.81 (0.70)	2.95 (0.73)	3.24 (0.85)	2	10.15^a^	**≤ 0.001**
CanTox: Euphoria/creativity	3.49 (0.72)	3.49 (0.68)	3.11 (0.96)	2	9.24^a^	**≤ 0.001**
Reward-craving	4.16 (1.84)	4.53 (1.81)	2.29 (1.63)	2	55.66^a^	**≤ 0.001**
Relief-craving	2.63 (1.51)	2.85 (1.35)	2.03 (1.35)	2	11.57^a^	**≤ 0.001**

CanTox, Cannabis-intoxication questionnaire; ^a^F-value; ^b^Chi^2^ value; df, degrees of freedom; Sig., level of significance; ^c^Employed, in training/unemployed; ^d^Diagnosis (yes/no/unknown).

Effect size for group comparisons regarding paranoia/dysphoria (*partial eta*^2^ = 0.13) indicates a large effect; regarding psychosis-like/loss of reality (*partial eta*^2^ = 0.094) it indicates a medium to large effect; regarding confusion/disorientation (*partial eta*^2^ = 0.044) it indicates a small to medium effect; regarding euphoria/creativity (*partial eta*^2^ = 0.040) it indicates a small to medium effect. The effect size for the group comparisons regarding reward-craving (*partial eta*^2^ = 0.20) indicates a large effect and regarding relief-craving (*partial eta*^2^ = 0.05) it indicates a medium effect. The effect size for the group comparisons regarding average frequency of cannabis use (*partial eta*^2^ = 0.02) indicates a small effect.

Post-hoc analyzes reveal significant group differences regarding paranoia/dysphoria between all groups: Past versus current users without intention to stop use (*p* ≤ 0.001), between past and current users with intention to stop use (*p* = 0.001) and between current users with versus without intention to stop use (*p* ≤ 0.001). Respective descriptive results, the past users reported the highest adverse effects, followed by users who intend to change their consumption. The lowest aversive effects reported current users without any intention to change use.

Significant group differences regarding psychosis-like/loss of reality were seen between past and both groups of current users (both *p* ≤ 0.001). Respective descriptive results, the past users reported higher adverse effects compared with current users.

Significant group differences regarding confusion/disorientation were also seen between past and both groups of current users (without intention to stop use: *p* ≤ 0.001; with intention to stop use: *p* = 0.007). Respective descriptive results, the past users again reported higher adverse effects compared with current users.

Significant group differences regarding positive intoxication effects, euphoria/creativity, were seen between past and both groups of current users (without intention to stop use: *p* = 0.001; with intention to stop use: *p* ≤ 0.001). Respective descriptive results, the past users reported lower positive effects compared with current users.

Regarding reward-craving, post-hoc analyzes revealed significant group differences between past and both groups of current users (both *p* ≤ 0.001). Respective descriptive results, past users reported less craving compared with current users.

Regarding relief-craving, past users were different from both groups of current users (without intention to stop use: *p* = 0.002; with intention to stop use: *p* ≤ 0.001). Respective descriptive results, past users reported less craving compared with current users.

Finally, respective average frequency of cannabis use, just both current users present significant different results (*p* = 0.028), due to the high standard deviations respective mean values.

### Qualitative information on reasons for abstinence in past users

Qualitative statements of past users on reasons for abstinence were assigned to seven thematic groups (multiple answers were allowed). In addition to the following presentation of the qualitative categories, a quantitative indication is given of how many of the respondents’ statements could be assigned to the corresponding category (in percent):

1. *Lack of energy*, such as sluggishness, fatigue, indifference, and unproductivity. Some example statements from subjects assigned to this category are: “*It makes you too indifferent*”; “*sluggishness in everyday life*”; “*listlessness*”; “*I could not get up for anything anymore*”; “*side effect lethargy and lack of drive*”. 41.7% of the statements were assigned to this category.
2. *Anxiety symptoms* Some example statements from subjects assigned to this category are: “*Anxiousness and self doubt, especially in groups*”; “*had anxiety too often*”; “*I had panic attacks*”. 25% of the statements were assigned to this category.
3. *Financial problems* Some example statements from subjects assigned to this category are: “*Costs too high*”; “*no more money*”; “*too expensive*”. 20% of the statements were assigned to this category.
4. *Neurocognitive deficits (concentration performance)* Some example statements from subjects assigned to this category are: “*Memory performance worsened*”; “*difficulty concentrating*”; “*memory loss*”. 12.2% of the statements were assigned to this category.
5. *Occupational problems* Some example statements from subjects assigned to this category are: “*When gliding as a pilot, smoking pot is bad*”; “*focusing on work was difficult*”; “*professional careers became more important*”. 12.1% of the statements were assigned to this category.
6. *Psychosis-like experiences* like the feeling, things would increasingly refer to oneself, or other people would look at them threateningly. Some example statements from subjects assigned to this category are: “*Impression, others are talking about me*”; “*feeling like I’m losing control, going crazy*”; “*paranoia, feeling like someone is there, following me*”. 10% of the statements were assigned to this category.
7. *Horror trip-intoxications* with still ongoing psychological consequences. Some example statements from subjects assigned to this category are: “*Horror trip and perceived start of psychosis*”; “*paranoia that then didn’t go away*”. 3.8% of the statements were assigned to this category.

## Discussion

We analyzed the predictive impact of cannabis intoxication effects on abstinence-motivational stages in an online sample. Potentially influencing parameters were included, such as craving, patterns of cannabis use and sociodemographics. Further analyzes compared past and current users (with and without intention to stop consumption) regarding outcome parameters with special focus on different intoxication effects.

Experiencing paranoia/dysphoria as a quality of cannabis intoxication was most predictive for abstinence motivation, especially for the higher motivational stages such as taking steps and recognition. Compared to other acute intoxication effects, paranoia/dysphoria also differentiated most clearly and with the highest effect size between the three groups: Among former users who retrospectively reported their past cannabis use experiences, paranoia and dysphoria were most pronounced, followed by present users with the intention to stop consumption. In present users without intention to stop cannabis use, paranoia and dysphoria seem to be least pronounced as acute intoxication effect.

Slightly less predictive for abstinence motivation was craving and male gender, as well as the two further negative acute intoxication effects (psychosis-like/loss of reality and confusion/disorientation), reaching significance only in the motivational stages recognition and ambivalence. Pleasant intoxication effects were not associated with abstinence motivation. These quantitative findings correspond with qualitative statements of past users: Lack of energy was the primary reason for abstinence, indicating a depressive symptom or part of the “a motivational syndrome”^44^. Lack of energy was followed by anxiety symptoms, both in turn corresponding with paranoia/dysphoria as quality of intoxication with the highest predictive impact for abstinence.

The less pronounced but nevertheless significant predictive impact of psychosis-like intoxication effects with respect to abstinence motivation indicated by our quantitative data corresponds with the 10–15% of persons qualitatively stating such experiences (PLE and “horror-trip”). This is in line with previous research stating that close to 15% of cannabis users report such experiences^29^.

The group comparison between current users (with versus without intention to quit use) versus past users revealed overall significant more unpleasurable intoxication effects in past users, whereas current users presented more pleasurable effects. Current users with versus without intention to quit were similar regarding most intoxication effects (apart from the above mentioned significant more paranoia/dysphoria within the intention to quit group). These findings highlight the importance of the specific quality of acute intoxication, especially with regard to the discussion of cannabis induced PLE, particularly emphasized in research literature^14^. In order to place the present findings comprehensibly in existing literature, the discussion regarding PLE must be elevated to the level of the individual items/symptoms. As previously criticized by colleagues^45^, the PLE-concept is poorly defined respective its content and time criteria. In the present operationalization, loss of reality and hallucinations are included in a dimension that we called the PLE/loss of reality-dimension (when beginning with our study). Other researchers present a dimension of the Cannabis Experiences Questionnaire, called “paranoia/dysphoria,” largely corresponding to the present dimension “paranoia/dysphoria”^10^. However, these authors refer to this dimension also as PLE, according to other groups stating that depressive and certain anxiety symptoms are intrinsic to the psychotic illness^45^.

Thus, we may consider now that it is essential to specify the dimensions of PLE. We may postulate that, in the sense of our research colleagues, we ultimately have two present PLE-dimensions: Besides our initially named PLE-dimension psychosis-like/loss of reality, also the dimension paranoia/dysphoria may be defined as part of a PLE-spectrum, the latter with even higher impact for abstinence or change motivation.

Corresponding, the PLE dimension in the study of Sami and colleagues^14^ integrates items that were presently factorized into the two so-called PLE-dimensions: PLE items of Sami and colleagues “fearfulness” and “feeling nervous” are assigned to the present paranoia/dysphoria dimension (present PLE-dimension 1), and PLE items of Sami and colleagues “feeling of going crazy”, “hearing voices” are assigned to the present psychosis like experiences/loss of reality dimension (present PLE-dimension 2).

Thus, respective its specific content, the present paranoia/dysphoria dimension (PLE 1) could be labeled as the “affective PLE dimension”, whereas the present PLE/loss of reality-dimension (PLE 2) could be labeled as the “perceptual PLE dimension” since it describes changes on the perceptual level, typically occurring in the context of hallucinogenic substances. Of course, it is extremely scary if psychotic experiences, such as hallucinations, occur in an individual, seemingly “out of nowhere” as part of a mental illness. However, some psychosis-like experiences are intentionally caused by some users of hallucinogenic substances such as psilocybin to alter consciousness^46^. Approximately 15.9% of the U.S. population older than 12 years ever used a hallucinogenic drug^46^. The dimension paranoia/dysphoria, however, describes affective changes towards a pole that may generally experienced as aversive. We suggest that presumably no person would take a substance with the a priori aim to induce paranoia and/or dysphoria. Instead, this is called a “horror trip” when it occurs, and according to the present qualitative evaluation, it represents a reason for cessation of consumption.

Regarding craving, it is remarkable that hedonistic reward craving is associated with the low motivation stage of ambivalence, whereas relief craving acts as a predictor at the next-highest stage of recognition. At the highest stage, taking steps, in which decisive steps of change have already taken place, craving is no longer significant. This suggests that craving for cannabis strongly decreases when users have succeeded in discontinuing consumption. But in current users, relief craving may be associated with some insight into problems caused by cannabis use, so that abstinence is considered.

In addition, gender was a predictor for all three motivational stages. Studies on cannabis predominantly examine men^47^. However, gender differences in cannabis use seem to exist. A research team found that women experience the effects of cannabis more pleasantly compared to men. Thus, women seem to be more receptive to further use than men after first use^48^. The present findings that male gender is associated with higher motivation to change cannabis use could be explained in this context. Therefore, if women experience cannabis effects as more pleasant on average, they may be less motivated to change use. However, the present data tend to indicate the opposite. Women differ from men with regard to acute intoxication effects only in that they experience fewer positive effects. An alternative explanation could be that men show more problematic patterns of use and thus have stronger subsequent problems, which then justify a motivation to change. This would be supported by the present data, with males using cannabis for significantly longer periods of time and showing shorter periods of abstinence than females. Other research data also point to stronger patterns of use among men. For example, Khan and colleagues report that men have longer episodes of cannabis use disorders compared to woman^49^, and Kerridge and colleagues report higher 12-month prevalences of cannabis use disorders in men^50^.

Furthermore, present data found that the number of abstinent weeks is negatively predictive associated with motivation to change. That is, the more regular the use, the more pronounced are the motivational stages of ambivalence and recognition. This fits with the finding of other researchers that higher severity of substance use predicts higher motivation to change^51^, as heavy use in particular will be associated with subsequent problems.

Finally, with regard to the clinical relevance of the present work, therapeutic approaches aiming to build up or maintain abstinence motivation could be highlighted on the one hand. Working on craving is common in the treatment of substance use disorders, for example in the dialectic behavioral therapy for substance use disorders. Here, craving is treated with anti-craving skills, similar to dealing with stress and inner tension^52^. Acute cannabis intoxication and the question of whether cannabis is fulfilling the user’s expectations fits well with the concept of motivational interviewing (MI). Our findings emphasize the importance of systematically integrating the acute intoxication effects into the training of MI professionals^53^.

In addition to the issue of cessation of cannabis use, another interesting question regarding intoxication effects is whether they are useful as predictors in the early detection of psychosis. This would make it possible to identify at-risk individuals among users, which would be a real gain for psychosis prevention. However, in order to increase the predictive value for psychosis, further criteria would probably have to be defined, such as the frequency of occurrence and their temporal persistence, as Schulze-Lutter and colleagues criticize the PLE-concept and in accordance with the risk symptoms attenuated psychotic symptoms (APS) and brief intermittent psychotic symptoms (BIPS)^45^. Another aspect that should be specified in the PLE concept is its content, i.e. the question of which symptoms are included and which are not. In this regard, the present study delivers a real benefit by differentiating PLE dimensions into the aforementioned affective and perceptual dimensions. We were at least able to show that these dimensions have different predictive values with regard to the motivation to change consumption. Further studies could investigate whether there is also a different predictive value with regard to cannabis use disorder and/or potential psychotic decompensation.

### Limitations

The primary limitation of our study is the cross-sectional design, which only allows a limited interpretation of a cause-and-effect relationship between the variables. Moreover, the available data are based on retrospective estimates and are therefore prone to recall errors. Another potential source of error is the use of self-report instruments, which are prone to certain perceptual errors. Regarding the question of present mental disorders, self-report is not very reliable compared to the use of established diagnostic instruments. Further, THC levels of cannabis used by subjects were not recorded, whereas Freeman and colleagues^12^ postulated an increased THC level in cannabis and other researchers^54^ reported increased psychosis-like intoxication effects at elevated THC levels. In addition, data on subjective intoxication effects may be biased because subjects retrospectively estimate a state in which they were acutely intoxicated. Moreover, the survey was conducted online. The prevailing pandemic corona situation did not allow for any other format. Thus, finally, it cannot be ruled out that individual participants provided implausible information, which would have been more noticeable in a face-to-face situation.

## Conclusions

Not all phenomena subsumed under cannabis induced psychosis-like experiences are experienced by users as aversive per se. Hallucinations, for example, can be fascinating, at least as long as they can be attributed to a hallucinogenic drug. Paranoia and dysphoria, on the other hand, are apparently unpleasant. This distinction proved crucial in the present study with regard to the predictive significance of PLE for abstinence motivation. Further studies should clarify the extent to which aversively experienced acute intoxication effects can be integrated into psychotherapeutic settings when working abstinence-oriented. In addition, the significance of certain cannabis-induced PLE should be investigated more closely with regard to the early detection of psychoses. For this purpose, however, PLE probably need to be specified by additional criteria, especially time criteria.

## Data Availability

The datasets generated during the current study are available from the corresponding author on reasonable request.
